# Definitive radiotherapy for uterine cervix cancer: long term results for patients treated in the period from 1998 till 2002 at the Institute of Oncology Ljubljana

**DOI:** 10.2478/raon-2013-0025

**Published:** 2013-07-30

**Authors:** Helena Barbara Zobec Logar, Barbara Segedin, Robert Hudej, Primoz Petric

**Affiliations:** Department of Radiotherapy, Institute of Oncology Ljubljana, Zaloška 2, 1000 Ljubljana, Slovenia;

**Keywords:** uterine cervix cancer, external beam radiotherapy, brachytherapy

## Abstract

**Background:**

The aim of this retrospective study was to analyse results of the two-dimensional (2D) uterine cervix cancer treatment at the Institute of Oncology Ljubljana from 1998 till 2002, before the three-dimensional (3D) approach was introduced in our clinical practice.

**Methods:**

Ninety-eight patients with the following FIGO stage distribution were analysed: 10% IB, 7% IIA, 37% IIB, 4% IIIA and 42% IIIB. The influence of age, haemoglobin level, histology, grade, stage, lymph node status, cumulative point A dose, and an overall treatment time on the survival and local control (LC) were evaluated. Acute and late side effects were assessed.

**Results:**

Five and 8-year overall survival (OS), disease specific survival (DSS) and LC rate were as follows: 47.2% and 43.0%, 54.7% and 53.4%, 74.9% and 72.5%, respectively. Point A dose and histology of the tumour influenced OS, positive lymph nodes DSS and point A dose LC rate. Probability of grade three and four late complications in the first five years was 7.1% for gastrointestinal tract and 3.3% for genitourinary system and vagina.

**Conclusions:**

Point A dose was independent predictor of OS and LC rate, lymph node status predicted DSS, while histology of the tumour influenced OS.

## Introduction

Brachytherapy (BT) in combination with external beam radiotherapy (EBRT) and chemotherapy plays a key role in the definitive treatment of locally advanced uterine cervix cancer.^1^–^6^

In the field of EBRT, sectional imaging has been widely implemented into the treatment planning process during recent decades. Three-dimensional (3D) conformal computed tomography (CT) based EBRT, employing megavoltage linear accelerators and customized shielding, nowadays represents a generally accepted approach to irradiation in the majority of tumours. Modern EBRT techniques, including intensity modulated radiotherapy and emerging new approaches, allow for increased dose conformity and a tight control over dose distribution in the irradiated tissues.

However, as far as gynaecological BT is concerned, treatment planning in the majority of institutions worldwide is currently still based on a two-dimensional (2D) approach, utilizing a geometrical system of points, defined on two orthogonal pelvic radiographs with the applicator in place.^6^–^11^ In cervix cancer BT, this approach refers mainly to dose prescription at point A^6^–^9^, while the dose to organs at risk (OAR) is most often reported at points as suggested by the International Commission on Radiation units and measurements (ICRU) Report 38 or their alternatives.^7^,^8^ Due to the absence of visual information on spatial interrelations between the applicator, the target volume and organs at risk, which differ from patient to patient, application to application and even within one application, the 2D approach is characterized by uncertainties regarding dose delivery to the irradiated tissues. In addition, the definition of point-related target and normal tissue dose constraints is controversial due to the steep dose gradient, dose inhomogeneity and non-contiguous high dose regions over the irradiated volume.^7^–^9^ It is therefore clearly more appropriate and reliable to correlate the effects of radiation on tissues with doses, absorbed in certain volumes, rather than at specific points.^12^–^18^ These correlations have been enabled by introduction of 3D sectional imaging into BT treatment planning.^12^–^14^,^17^

Nevertheless, a large amount of evidence that supports the correlation between conventional radiography based point doses and the clinical outcome exists.^7^,^8^,^12^–^14^,^17^–^25^

At the Institute of Oncology Ljubljana we have been performing 2D radiography based uterine cervix cancer BT until 2006. Subsequently, 3D MRI assisted BT treatment planning has been systematically implemented and currently represents our standard treatment approach.^26^,^27^ When introducing a new method of planning, prescribing, recording and reporting the treatment, it is essential to have a thorough knowledge and understanding of existent traditional institutional techniques. Only by a careful analysis of long standing experience and by linking conventional dosimetric and clinical parameters to the sectional imaging based data, it is possible to avoid potentially hazardous deviations from 2D approach and to fully exploit the benefits of 3D sectional imaging based BT.

This report summarizes a single institutional experience with 2D radiography based treatment planning in uterine cervix cancer radiotherapy in a time period between 1998 and 2002. After 2002, 3D conformal CT-based EBRT was systematically introduced in our clinical practice.

## Patients and methods

### Patients and tumours

Ninety-eight patients with histologically confirmed uterine cervix cancer and complete medical records that were treated with curative intent with radiotherapy +/− chemotherapy at the Institute of Oncology Ljubljana in the period from January 1998 till December 2002 were enrolled and retrospectively analysed. The investigators followed recommendations of the Helsinki Declaration (1964, with later amendments) and of the European Council Convention on Protection of Human Rights in Bio-Medicine (Oviedo 1997).

Mean patient age was 60.1 (23 to 85) years. Pre-treatment patient work-up consisted of gynaecological examination (all patients), chest radiography (98%), cystoscopy (42%), proctoscopy (32%), intravenous urography (19%), abdominal ultrasound (45%) and computed tomography (17%). Each patient was examined by at least three independent examiners, two gynaecologists and one radiation oncologist.

FIGO stage distribution was as follows: 10% IB, 7% IIA, 37% IIB, 4% IIIA and 42% IIIB.

The predominant histological type was squamous cell carcinoma (SCC), representing 92% of all tumours, followed by adenocarcinoma (3%) and other histologies (5%). The tumours were well, moderately and poorly differentiated in 10, 25 and 30%, respectively.

### Treatment

The mean interval between the first presentation and the beginning of the treatment was 24 days (range 1–60). All patients were treated with a combination of EBRT +/− concurrent chemotherapy and BT. Mean overall treatment time was 52 days ± 11 days.

EBRT was delivered following conventional radiography-based simulation at a 5–15 MV linear accelerator in 99% and at a Cobalt-60 device in 1% of patients. A mean dose applied via pelvic fields was 40 Gy (20–60 Gy) (2 Gy per fraction, five fractions weekly). A four field technique was applied in 10% and the technique of two opposite fields in 90%.

Paraaortic radiotherapy (mean dose: 32 Gy; range: 20–40 Gy) was carried out in four (4.1%) patients with positive paraaortic (n = 3) and interiliac (n = 1) lymph nodes, as determined by ultrasound or CT.

Six (6.0%) patients received chemotherapy (weekly cisplatin, 40 mg/m^2^) concurrent with EBRT.

Following completion of EBRT, low dose rate (LDR) brachytherapy with ^137^Cs source was applied. A Henschke-type metallic applicator consisting of an intrauterine tandem and a pair of vaginal ovoids was utilized. In 95 (97%) patients, one insertion was performed. Three (3%) patients received two insertions after an interval from one to four weeks. Following insertion, two orthogonal pelvic radiographs with the applicator in place were taken. A mean nominal dose of 25 Gy (range 12–36 Gy) was prescribed to point A. Treatment planning was carried out using our in-house developed software application, and was based on the information obtained from the orthogonal radiography. Nominal doses at the ICRU-points for the rectum (ICRU-R) and bladder (ICRU-B) were calculated and recorded.^8^

For the purpose of adding doses from EBRT and BT, it was assumed that the EBRT dose to Manchester point A, point B (3 cm lateral to point A) and to the ICRU points for the bladder and rectum equalled the prescribed EBRT dose. In this study, at time of BT, the rate of dose delivery by the ^137^Cs source was not equal to the reference dose rate (0.5 Gy/h). In addition, the dose rate was not equal for all patients due to a gradual decrease of ^137^Cs source activity with time. Therefore, to enable meaningful comparisons of individual treatments, cumulative (EBRT + BT) biologically equivalent doses (EQD2) to the Manchester point A, point B and ICRU points for the bladder and rectum were calculated using the linear quadratic model (reference EBRT dose per fraction = 2 Gy, reference BT dose rate = 0.5 Gy/h, α/β for the tumour = 10 Gy, α/β for the organs at risk = 3 Gy, sublethal damage repair half time = 1.5h).^7^ During treatment planning, we aimed at keeping the biological equivalent dose at the ICRU-R and ICRU-B points below our departmental limits of 70 Gy. No attempt was made to assess the dose to other organs at risk (*i.e*. sigmoid colon, small bowel and vagina).

### Follow up

Acute treatment side effects were assessed during the treatment and recorded descriptively in the patient chart. For the purpose of this study the Radiation Therapy Oncology Group (RTOG) criteria^28^ were employed in an attempt to retrospectively assign corresponding RTOG toxicity levels to individual cases, according to the descriptions in the patient charts. The haemoglobin level was recorded before, during and after the treatment.

Chronic side effects were evaluated at the time of each follow-up visit after the treatment and are reported here according to LENT-SOMA scale.^29^ Follow-up investigations were performed respecting our institutional guidelines.^30^–^32^

Overall survival (OS), disease specific survival (DSS) and local control (LC) actuarial rates were defined as the period from the date of biopsy to the date of death, disease related death and first documented evidence of disease progression or recurrent tumour in true pelvis, respectively.

### Statistical analysis

The data were analysed using SPSS 13.0 statistical software package (version 13 for Windows, Copyright© SPSS Inc., Chicago, Illinois). All statistical tests were double-sided; differences at p < 0.05 were considered statistically significant.

T-test was used to assess the statistical significance of differences between values of continuous variables. Kaplan-Meier method was applied to calculate actuarial survival and LC rates. Patients without recurrence were censored at time of the last follow-up, visit or death. Surviving patients were censored at time of the last follow-up. Frequencies of different grades of acute and chronic toxicity were calculated. Using the univariate statistical analysis, the influence of FIGO stage, age, nodal status, haemoglobin level, histological type, grade, cumulative EQD2 at point A and overall treatment time on the survival and LC rates were assessed by using a log-rank test. Variables such as age, haemoglobin level and overall treatment time were analysed as quantitative variables using arbitrary cut points (Hb < 100 g/l, Hb ≥ 100 g/l, overall treatment time ≤ 50 days and > 50 days, age < 60, ≥ 60 years). A multivariate analysis, based on the Cox form of the proportional hazards regression model, was performed to test possible predictive variables (method enters probability to enter 0.05, probability to remove 0.1).

## Results

### Dosimetric data

**FIGURE 1.**
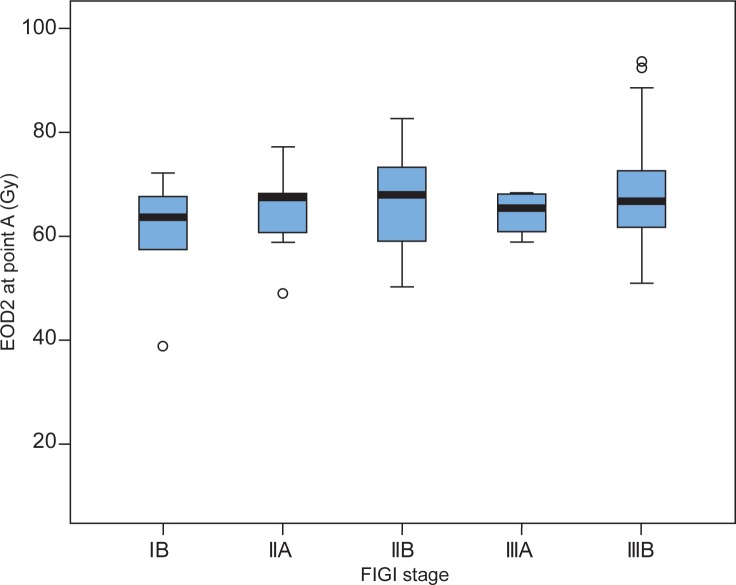
Cumulative EQD2 delivered at point A in different FIGO stages. Black horizontal lines represent mean values of EQD2 for each FIGO stage. The height of the box is equal to the interquartile range (IQR), which is the range within which the middle 50% of the ranked data are found. The whiskers indicate the range of the data. Dots represent extreme values.

Cumulative (EBRT + BT) EQD2 at Manchester point A, B, ICRU-R and ICRU-B points are presented in Table 1. There were no statistically significant differences in the mean EQD2 delivered at point A between patients with different FIGO stages (Figure 1). The point A EQD2 was below 60 Gy in 26.0 %, between 60 and 80 Gy in 70.0 % and above 80 Gy in 5.0% of patients, respectively. The mean cumulative EQD2 at ICRU-B and ICRU-R point was 56.9 Gy (range 37.5–79.3) and 62.2 (range 38.3–82.5), respectively.

### Survival and local control

**FIGURE 2.**
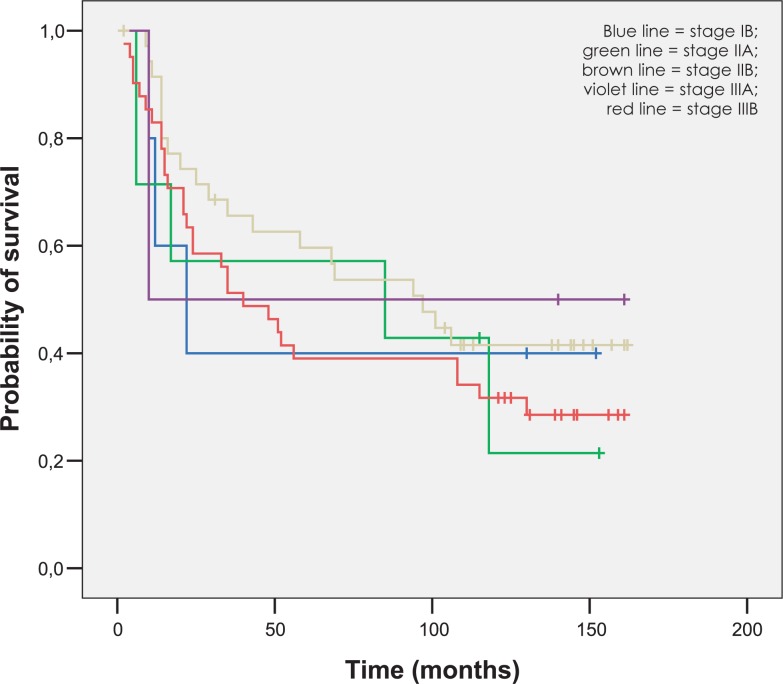
Overall survival according to FIGO stage.

**FIGURE 3.**
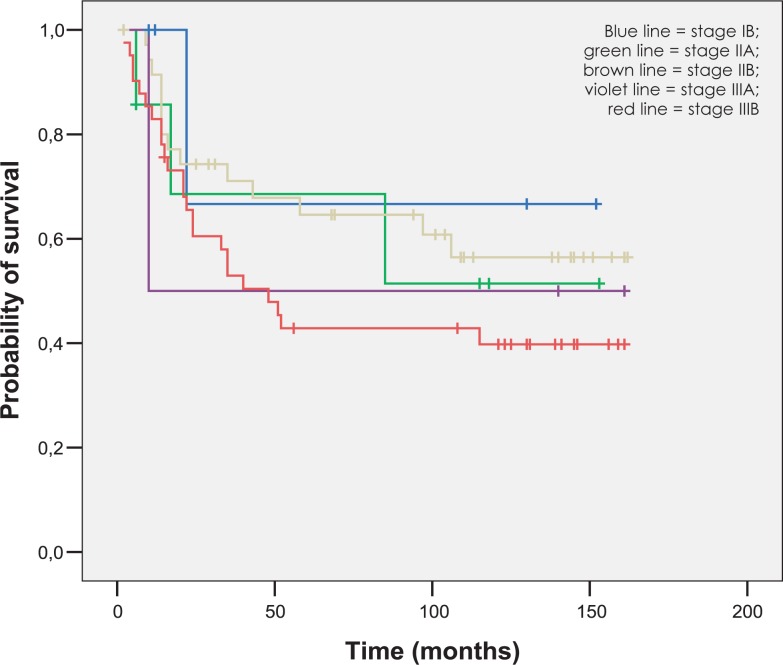
Disease specific survival according to FIGO stage.

**FIGURE 4.**
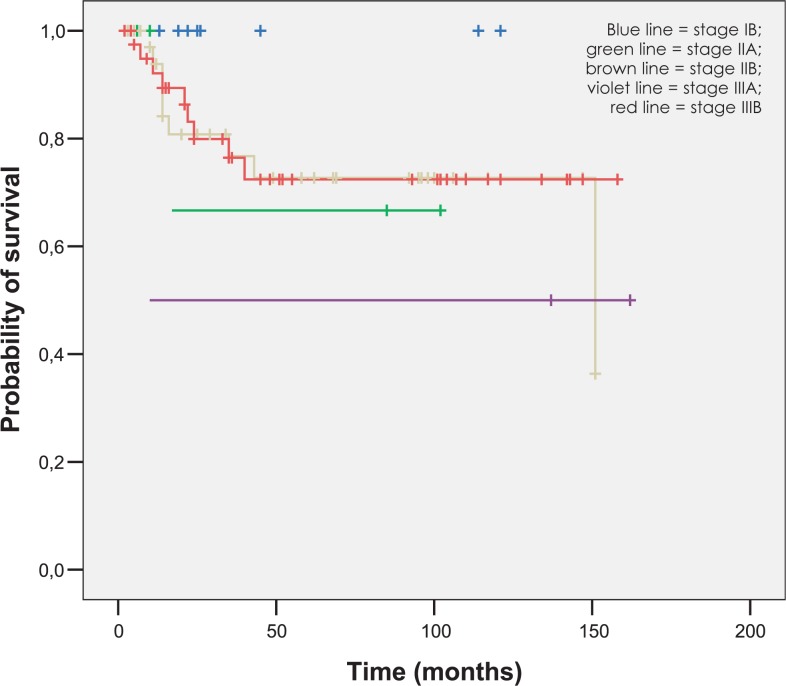
Local control according to FIGO stage.

The mean follow-up was 77 months (range 2–162 months). The 5-year OS and DSS rates were 47.2% and 54.7%. As expected, the 5-year OS rate was significantly lower for metastatic disease (patients with positive paraaortic lymph nodes and distant relapse), as for nonmetastatic disease (5.6% *versus* 54.7%, p = 0.000). OS, DSS and LC rates for different FIGO stages at five end eight years are represented in Figures 2 to 4.

The overall LC rate at five years was 74.9%. Six out of 98 patients (6%) had residual disease at the first follow-up (2 stage IIB, 1 stage IIIA and 3 stage IIIB). Two patients developed distant metastases during the treatment (both stage IIIB). All of these patients died.

Twenty-seven (28.0%) out of 98 patients developed a relapse, 11 (11%) in pelvis, 15 (15%) at distant sites and one (1%) in pelvis and distant sites simultaneously. A central pelvic relapse was observed in eight (8%) and a side wall relapse in three patients (3%). Seven patients were secondarily treated with salvage surgery or radiotherapy, but none of them survived.

The first sites of distant relapse were paraaortic lymph nodes in six, lung in four, liver in three, supraclavicular nodes in two and bone in one patient. For patterns of recurrence see Table 2.

The overall LC rate at eight years was 72.5%. After eight years of the follow up one more stage IIA patient developed a central pelvic relapse. One stage IIIB patient developed a distant relapse (paraaortic lymph nodes and lung) after 9.2 years of the follow up.

All three patients with positive paraaortic lymph nodes died within 14 months from the time of diagnosis. The only patient with enlarged lymph nodes, who survived to the time of analysis, was the one with positive interiliacal nodes.

**FIGURE 5.**
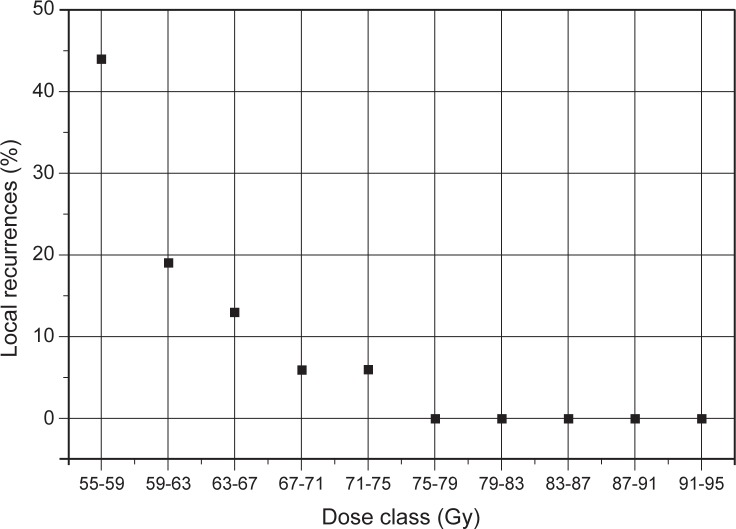
Proportion of local recurrences as a function of the point A dose. Point A dose is divided in ten dose classes with a class interval of 4 Gy.

Point A dose had an important role in LC and survival. The 5-year OS rate was 32.1% if the dose to point A was less than 65 Gy and 56.3% when point A dose was 65 Gy or more (p = 0.005). The same was true for DSS and LC. The 5-year DSS was 48.4% for the lower dose and 69.1% for the higher dose (p = 0.08). LC rate was 40.2% for the lower dose versus 84.0% for the higher dose (p = 0.03). The proportion of local recurrences was lower with a higher point A dose (Figure 5). Above 75 Gy no local recurrence was registered.

The histological type of the tumour influenced OS, which was better for SCC than for adenocarcinoma or adenosquamous carcinoma (46.3 *vs.* 0%; p ≤ 0.005). Positive lymph nodes were associated with a drop in 5-year DSS. If the lymph nodes were negative DSS was 64.5%, vs. 18.8% in case of positive iliac or paraaortic lymph nodes (p = 0.01).

Variables such as age, grade, stage, haemoglobin level and overall treatment time didn’t influence survival and LC.

In the multivariate analysis, point A dose retained its independent prognostic value for the OS (p = 0.03, hazard ratio (HR) = 0.5, confidence interval (CI) = 0.3–0.9).

### Acute and late side effects

Acute gastrointestinal toxicity was reported in twelve (12.2%) patients. In general it was mild, with grade 1, 2 and 3 proctitis appearing in eight (8.3%) two (2.0%) and two (2.0%) patients, respectively. Acute urinary side effects were reported only in four (4.0%) patients, grade 1 in two (2.0%), and grade 3 in one (1.0%) patient. Only one patient (1.0%) experienced grade 4 acute toxicity.

The mean initial haemoglobin level was 120 (82–152) g/l, fell to 111 (59–150) g/l during the treatment and rose again after the treatment to a mean level of 120 (83–155) g/l in the next four months. The difference between initial and nadir haemoglobin levels was statistically significant (p < 0.005). Transfusion received 14.9% of the patients and erythropoietin received 3.1% of the patients on chemotherapy. There was no statistically significant difference in survival and LC in patients with a low haemoglobin level.

Chronic gastrointestinal side effects were reported by fifteen (15.3%) patients with eight (8.2%) experiencing grade 1, one (1.0%) grade 2, two (2.0%) grade 3 and four (4.1%) grade 4 late gastrointestinal toxicity. One patient developed a rectovaginal fistula, two patients developed ileus, *anus praeter* was formed in one patient.

Ten (10.2%) patients experienced late genitourinary tract toxicity: four (4.1%) grade 1, four (4.1%) grade 2 and two (2.0%) grade 4. Incontinence, haematuria, rise of serum creatinine level and mild hydronephrosis were the most common chronic genitourinary side effects. Rise of creatinine level and mild hydronephrosis developed independently of chemotherapy.

Twelve (12.2%) patients experienced late vaginal toxicity, five (5.1%) grade 1, five (5.1 %) grade 2, one (1.0%) grade 3 and one (1.0%) grade 4. Vaginal stenosis represented the most commonly reported complication.

The probability of developing late side effects of any grade in first five years was 16.6% for gastrointestinal tract, 15.7% for genitourinary system and 22.3% for the vagina. The probability of grade three and four late side effects in the first five years was 7.1% for gastrointestinal tract and 3.3% for genitourinary system and vagina.

The mean interval before developing the late complication of any grade was 27.6 months (range 6–96 months) for gastrointestinal, 28.7 months (range 3–65 months) for genitourinary and 27.2 months (range 5–53 months) for late vaginal squeal. The mean interval before developing a serious late complication (grade 3 and 4) was 26.5 (range 3–94 months) for gastrointestinal tract, 21.5 months (range 16–27) for genitourinary tract and 21.5 months (range 16–27 months) for the vaginal complications.

## Discussion

In the present study we evaluated historical data of combined EBRT and 2D LDR BT in the treatment of uterine cervix cancer. All the data were collected in a retrospective manner and only patients who received combined EBRT and BT treatment were included. There was no stage IVA disease patient treated with a combined therapy in the study. The weakness of the study was also uneven distribution of patients in different FIGO stages, especially a low number of stage IIA and IIIA patients, which was less than 10%.

The 5-year OS and DSS rates for all 98 patients were 47.2% and 54.7%. OS rate for stage IB was only 40.0%, which was lower than expected and lower than the reported survival rates for more advanced disease.^33^–^39^ Mean age of patients with stage IB disease was higher when compared to the whole group (68.8 ±13.4 years compared to 60.1±14.2 years), so other age related comorbidity factors may have caused lower OS rate. In five out of ten patients in stage IB the death was not associated with primary disease, one patient died of breast cancer, two of metastatic disease due to uterine cervix cancer.

Five-year OS and DSS rates for stage IIA were 44.0% and 55.6%, with LC rate of 66.7%. Low LC rate (50.0%) was also observed in stage IIIA with DSS rate of 50.0%. The Vienna group reported 100.0% and 52.7% pelvic control rate for stage IIA and IIIA at three years, respectively, while Perez *et al*. reported a pelvic failure rate of 0–28% in stage IIA disease and 32–50% in stage III disease at ten years.^35^,^38^ Barillot *et al.* reported 14.5% 5-year pelvic failure rate in stage IIA disease and 40% in stage IIIA disease.^39^ We believe that rather high pelvic failure rates in these stages (33.3% in stage IIA, 50.0% in stage IIIA), were mainly due to the extension of the disease in the vagina and poor coverage of the primary tumour with the standard applicator at BT. This could have resulted in lower DSS and OS rates as it would be expected for these two stages.^33^–^39^ Also the number of patients in both stages (seven in stage IIA, four in stage IIIA) was not big enough to make any definite conclusions.

LC rate in stage IIIB group, also the largest group of patients, was very good (72.4%). The Vienna group reported 69.1% LC rate at three years, while Ferrigno *et al.* reported 62 % in stage II and III patients and 58% in stage III at five years.^35^,^40^,^41^ In the study of Barillot *et al*. the incidence of local failure at five years in stage IIIB was 48.5%.^39^ The possible reason for high LC in stage IIIB and low LC rate in other stages (IIA and IIIA) can be explained by the lack of diagnostic procedures (CT and MRI) used to determine TNM stage.

Point A dose showed to be an important factor determining not only LC rate but also OS rate. If the dose was lower than 65 Gy, the 5-year survival rate was 32.2% and LC rate 59.8%, on the other hand if the dose was equal or higher than 65 Gy, OS was 55.1 % and LC rate as high as 89.2%. A rather high LC rate can be partly explained by the lack of stage IVA patients in the study. The Vienna group reported on OS and pelvic control rate at three years of 40% and 60% for stage IVA, and of 58.2% and 77.6% for all patients, respectively.^35^ Perez *et al.* included 20 patients in stage IVA disease. There were no-long term survivors among them. The overall incidence of pelvic recurrences in stage IVA was 72%, and of distant metastases 55%, respectively.^38^ Barillot *et al.* reported 5-year OS rate of 23% and local failure rate of 100% in stage IV, and of 68% in all stages, respectively.^39^

Dose is an important factor for the tumour cure. The optimal dose for the tumour is balanced with the dose to critical organs - sigmoid colon, rectum and bladder. The mean dose to point A in our patients was 66.5 Gy, while the mean dose to organs at risk was below our departmental constraints. Gastrointestinal late toxicities developed in 15.3%, 6.1% were grade 3 and 4 late reactions. Probability of grade three and four late side effects in the first five years was 7.1% for gastrointestinal tract. This is comparable with the Vienna group, which reported the incidence of 6.1% at three years.^35^ Lorvidhaya reported 7% combined grade 3 and 4 late complication rate for bowel and bladder.^42^ Based on our own experience with MRI based planning in BT and according to the literature, the dose to the rectum (D2cc) is usually lower than the dose to other critical organs.^12^,^13^,^27^,^43^,^44^ Most of the published series report a higher dose to the sigmoid colon than to the rectum. In other words, late gastrointestinal toxicities could be mainly correlated to the high dose to the sigmoid colon rather than to the dose to the rectum.^45^ In historical cases the dose to the sigmoid was not registered, so the correlation between the dose to the rectum and late toxicities is not straightforward. However, many studies that compared ICRU doses to critical organs and dose-volume histogram parameters (D2cc) proved a good correlation between ICRU dose and D2cc, especially for the rectal ICRU dose.^46^–^49^

Serious late bladder complications (grade 3 and 4) were rather rare and reported only in 2% of patients. Vaginal late toxicities grade 3 and 4 were also not as common as reported in the literature, probably because they were not systematically and prospectively recorded.^50^ Vaginal late side effects are not commonly reported in the literature.^39^,^42^ The Vienna group is one of the few who provide data on vaginal morbidity after definitive radiotherapy. They reported a 30.6% grade 3 and 4 vaginal complication rate.^35^

Fifteen out of 27 patients with recurrent disease developed relapse at distant sites. One of the reasons for a big proportion of distant failures could be, that CT/MRI were not yet systematically used in uterine cervix cancer patients at that time and only a minority of patients (6%) received concurrent chemotherapy with EBRT. The causes for omitting chemotherapy were advanced age, hydronephrosis, afunction/hypofunction of one or both kidneys, raised creatinin level and other comorbidities. A more systematic introduction of concurrent chemotherapy at our Institute started only after the year 2000.^1^ It can be speculated that with the more wide implementation of sensitizing chemotherapy into the primary treatment of advanced disease distant failures can be further reduced and the survival improved.^50^–^53^ Histology, point A dose and presence of positive lymph nodes proved to be critical factors that affect prognosis. Other variables such as age, grade, stage, overall treatment time and tumour size also influenced the survival and LC in other studies.^38^,^39^,^54^ Their relevance was not confirmed in our study. The tumour size was not tested as the covariate in this study because it was not systematically monitored.

## Conclusions

Results of the combined treatment (conventional 2D EBRT + LDR BT) of uterine cervix cancer at the Institute of Oncology Ljubljana were evaluated. 2D based BT approach was associated with good rates of LC in stage IIIB disease, which was the largest group in our study. Variables, influencing prognosis were histology, point A dose and lymph node status. Improved LC and reduced morbidity rates may be expected in the era of the systematic implementation of MRI-based adaptive BT at our department. Treatment results could be further improved with the development of new (individually tailored) applicators to enable the adequate dose-coverage of advanced tumours.

## Figures and Tables

**TABLE 1.** Cumulative (EBRT + BT) biologically equivalent doses (EQD2) at Manchester point A, point B and ICRU points for the bladder and rectum (ICRU-B and ICRU-R)

**Point**	**EQD2 mean [range] (GY)**
A	66.5 [38.7 – 93.6]
B	60.1 [24.2 – 69.0]
ICRU-B	56.9 [37.5 – 79.3]
ICRU-R	62.2 [38.3 – 82.5]

**TABLE 2.** Pattern of progression and relapse according to FIGO stage. The numbers represent numbers of patients which equals to per cent of patients

**Stage**	**IB**	**IIA**	**IIB**	**IIIA**	**IIIB**	**Total**
**Total number**	10	7	36	4	41	98
**Local progression**			2	1	3	6
**Distant progression**					2	2
**Pelvic relapse (central/ side wall)**		1 (1/0)	4 (3/1)	1 (1/0)	5 (3/2)	11 (8/3)
**Distant relapse**	2	1	4	1	7	15
**Pelvic and distal relapse**			1			1
**Overall failure**	2	2	11	3	17	35
**No evidence of disease**	8	5	25	1	24	63

## References

[1] Morris M, Eifel PJ, Lu J, Grigsby PW, Levenback C, Stevens RE (1999). Pelvic radiation with concurrent chemotherapy compared with pelvic and para-aortic radiation for high-risk cervical cancer. N Engl J Med.

[2] Keys HM, Bundy BN, Stehman FB, Mudersprach LI, Chafe WE, Suggs CL (1999). Cisplatin, radiation, and adjuvant hysterectomy for bulky stage IB cervical carcinoma. N Engl J Med.

[3] Whitney CW, Sause W, Bundy BN, Malfetano JH, Hannigan EV, Fowler WC (1999). Randomized comparison of fluorouracil plus cisplatin versus hydroxyurea as an adjunct to radiation therapy in stage IIB-IVA carcinoma of the cervix with negative para-aortic lymph nodes: a Gynecologic Oncology Group and Southwest Oncology Group study. J Clin Oncol.

[4] Green JA, Kirwan JM, Tierney JF, Symonds P, Fresco L, Collingwood M (2001). Survival and recurrence after concomitant chemotherapy and radiotherapy for cancer of the uterine cervix: a systematic review and meta-analysis. Lancet.

[5] Vale C (2008). Reducing uncertainties about the effects of chemoradiotherapy for cervical cancer: A systematic review and meta-analysis of individual patient data from 18 randomized trials. J Clin Oncol.

[6] Gerbaulet A, Pötter R, Haie-Meder C, Gerbaulet A, Pötter R, Haie-Meder C (2002). Cervix cancer. The GEC ESTRO handbook of brachytherapy.

[7] Pötter R, Van Limbergen E, Wambersie A, Gerbaulet A, Pötter R, Mazeron JJ, Meertens H, Van Limbergen E (2002). Reporting in brachytherapy: dose and volume specification. The GEC ESTRO handbook of brachytherapy.

[8] ICRU International Commission of Radiation Units and Measurements (1985). Dose and volume specification for reporting intracavitary therapy in gynaecology. ICRU Report 38.

[9] Massey JB, Pointon RS, Wilkinson JM (1985). The Manchester System and the BCRU recommendations for brachytherapy source specification. Br J Radiol.

[10] Guedea F, Ellison T, Venselaar J, Borras JP, Hoskin P, Poetter R (2007). Overview of brachytherapy resources in Europe: a survey of patterns of care study for brachytherapy in Europe. Radiother Oncol.

[11] Guedea F, Ellison T, Hoskin P, Hellebust TP, Didier Peiffert D, Londres B (2010). Patterns of care of brachytherapy in Europe: updated results. Radiother Oncol.

[12] Haie-Meder C, Pötter R, Van Limbergen E, Briot E, De Brabandere M, Dimopoulos J (2005). Recommendations from Gynaecological (GYN) GECESTRO Working Group (I): concepts and terms in 3D image based 3D treatment planning in cervix cancer brachytherapy with emphasis on MRI assessment of GTV and CTV. Radiother Oncol.

[13] Pötter R, Haie-Meder C, Van Limbergen E, Briot E, De Brabandere M, Dimopoulos J (2006). Recommendations from gynaecological (GYN) GEC ESTRO working group (II): concepts and terms in 3D image-based treatment planning in cervix cancer brachytherapy-3D dose volume parameters and aspects of 3D image-based anatomy, radiation physics, radiobiology. Radiother Oncol.

[14] Wachter-Gerstner N, Wachter S, Reinstadler E, Fellner C, Knocke TH, Wambersie A (2003). Bladder and rectum dose defined from MRI based treatment planning for cervix cancer brachytherapy: comparison of dose-volume histograms for organ contours and organ wall, comparison with ICRU rectum and bladder reference point. Radiother Oncol.

[15] Pötter R, VanLimbergen E, Gerstner N, Wambersie A (2001). Survey of the use of the ICRU 38 in recording and reporting cervical cancer brachytherapy. Radiother Oncol.

[16] Pötter R, Gerbaulet A, Pötter R, Mazeron JJ, Meertens H, Van Limbergen E (2002). Modern imaging in brachytherapy. The GEC ESTRO handbook of brachytherapy.

[17] Barillot I, Horiot JC, Maingon P, Truc G, Chaplain G, Comte J (2000). Impact on treatment outcome and late effects of customized treatment planning in cervix carcinomas: baseline results to compare new strategies. Int J Radiat Oncol Biol Phys.

[18] Perez CA, Grigsby PW, Lockett MA, Chao KS, Williamson J (1999). Radiation therapy morbidity in carcinoma of the uterine cervix: dosimetric and clinical correlation. Int J Radiat Oncol Biol Phys.

[19] Kovalic JJ, Perez CA, Grigsby PW, Lockett MA (1991). The effect of volume of disease in patients with carcinoma of the uterine cervix. Int J Radiat Oncol Biol Phys.

[20] Perez CA, Fox S, Lockett MA, Grigsby PW, Camel HM, Galakatos A (1991). Impact of dose in outcome of irradiation alone in carcinoma of the uterine cervix: analysis of two different methods. Int J Radiat Oncol Biol Phys.

[21] Roeske JC, Mundt AJ, Halpern H, Sweeney P, Sutton H, Powers C (1997). Late rectal sequelae following definitive radiation therapy for carcinoma of the uterine cervix: A dosimetric analysis. Int J Radiat Oncol Biol Phys.

[22] Chen SW, Liang JA, Yeh LS, Yang SN, Shiau AC, Lin FJ (2004). Comparative study of reference points by dosimetric analyses for late complications after uniform external radiotherapy and high-dose-rate brachytherapy for cervical cancer. Int J Radiat Oncol Biol Phys.

[23] Ogino I, Kitamura T, Okamoto N, Yamasita K, Aikawa Y, Okajima H (1995). Late rectal complication following high dose rate intracavitary brachytherapy in cancer of the cervix. Int J Radiat Oncol Biol Phys.

[24] Ferrigno R, dos Santos Novaes PE, Pellizzon AC, Maia MA, Fogarolli RC, Gentil AC (2001). High-dose-rate brachytherapy in the treatment of uterine cervix cancer. Analysis of dose effectiveness and late complications. Int J Radiat Oncol Biol Phys.

[25] Kim HJ, Kim S, Ha SW, Wu HG (2008). Are doses to ICRU reference points valuable for predicting late rectal and bladder morbidity after definitive radiotherapy in uterine cervix cancer?. Tumori.

[26] Petric P, Hudej R, Marolt-Mušič M (2009). MRI assisted cervix cancer brachytherapy pre-planning, based on insertion of the applicator in para-cervical anaesthesia: preliminary results of a prospective study. J Contemp Brachytherapy.

[27] Petric P, Hudej R, Rogelj P, Blas M, Segedin B, Zobec Logar HB, Dimopoulos JCA (2012). Comparison of 3D MRI with high sampling efficiency and 2D multiplanar MRI for contouring in cervix cancer brachytherapy. Radiol Oncol.

[28] Cox JD, Stetz J, Pajak TF (1995). Toxicity criteria of the Radiation Therapy Oncology Group (RTOG) and the European Organization for Research and Treatment of Cancer (EORTC). Int J Radiat Oncol Biol Phys.

[29] (1995). LENT SOMA scales for all anatomic sites. Int J Radiat Oncol Biol Phys.

[30] Baškovič M, Cerar O, Kaučič M, Kocijan A, Kovačič J, Kuhelj J (1992). [Guidelines for the treatment of the gynaecological malignancies]. [Slovenian].

[31] Baškovič M, Bebar S, Cerar O, Fras AP, Kragelj B, Robič V (2001). [Gynaecological malignancies. Guidelines for the treatment at the Institute of Oncology Ljubljana and Gynaecological Clinic of the University Medical Centre Ljubljana]. [Slovenian].

[32] Stržinar V, Baškovič M, Bebar S, Cerar O, Fras AP, Koritnik K (2002). [Gynaecological malignancies. Guidelines for the treatment at the of Oncology Ljubljana and Gynaecological Clinic of the University Medical Centre Ljubljana]. [Slovenian].

[33] Perez Ca, Breaux S, Bedwinek JM, Madoc-Jones H, Camel HM, Purdy JA (1984). Radiation therapy alone in the treatment of carcinoma of the uterine cervix. II. Analysis of complications. Cancer.

[34] Fletcher GH, Hamburger AD, Fletcher GH (1980). Female pelvis Squamous cel carcinoma of the uterine cervix. Textbook of radiotherapy.

[35] Pötter R, Knocke TH, Fellner C, Baldass M, Reinthaller A, Kucera H (2000). Definitive radiotherapy based on HDR brachytherapy with iridium 192 in uterine cervix carcinoma: report on the Vienna University Hospital findings (1993–1997) compared to the preceding period in the context of ICRU 38 recommendations. Cancer Radiother.

[36] Horiot JC, Pigneux J, Pourquier H (1988). Radiotherapy alone in carcinoma of the intact uterine cervix according to G. H. Fletcher guidelines: a French cooperative study of 1383 cases. Int J Radiat Oncol Biol Phys.

[37] Pernot M, Hoffstetter S, Peiffert D, Carolus JM, Guillemin F, Verhaeghe JL (1995). Statistical study of a series of 672 cases of carcinoma of the uterine cervix. Results and complications according to age and modalities of treatment. Bull Cancer.

[38] Perez CA, Grigsby PW, Chao KS, Mutch DG, Lockett MA (1998). Tumor size, irradiation dose, and long-term outcome of carcinoma of uterine cervix. Int J Radiat Oncol Biol Phys.

[39] Barillot I, Horiot JC, Pigneux J, Schraub S, Pourquier H, Daly N (1997). Carcinoma of the intact uterine cervix treated with radiotherapy alone: a French cooperative study: update and multivariate analysis of prognostics factors. Int J Radiat Oncol Biol Phys.

[40] Ferrigno R, dos Santos Novaes PE, Pellizzon AC, Maia MA, Fogarolli RC, Gentil AC (2001). High-dose brachytherapy in the treatment of uterine cervix cancer. Analysis of dose effectiveness and late complications. Int J Radiat Oncol Biol Phys.

[41] Ferrigno R, Campos de Oliveira Faria SL, Weltman E, Salvajoli JV, Segreto RA, Pastore A (2003). Radiotherapy alone in the treatment of uterine cervix cancer with telecobalt and low-dose-rate brachytherapy: retrospective analysis of results and variables. Int J Radiat Oncol Biol Phys.

[42] Lorvidhaya V, Tonusin A, Changwiwit W, Chitapanarux I, Srisomboon J, Wanwilairat S (2000). High-dose-rate afterloading brachytherapy in carcinoma of the cervix: an experience of 1992 patients. Int J Radiat Oncol Biol Phys.

[43] Dimopolus J, Schard G, Berger D, Lang S, Goldner G, Helbich T (2006). Systematic evaluation of MRI findings in different stages of treatment of cervical cancer: potential of MRI on delineation of target, patho-anatomical structures and organs at risk. Int J Radiat Oncol Biol Phys.

[44] Kirisits C, Lang S, Dimopoulos J, Berger D, Georg D, Pötter R (2006). The Vienna applicator for combined intracavitary and interstitial brachytherapy of cervical cancer: Design, application, treatment planning, and dosimetric results. Int J Radiat Oncol Biol Phys.

[45] Erickson B, Grossheim L, Mai J (2004). Imaging the nondisplaced rectosigmoid during HDR brachytherapy for cervical cancer can alter dose specification. [Abstract]. Radiother Oncology.

[46] Pelloski CE, Palmer M, Chronowski GM, Jhingran A, Horton J, Eifel PJ (2005). Comparison between CT-based volumetric calculations and ICRU reference-point estimates of radiation doses delivered to bladder and rectum during itracavitary radiotherapy for cervical cancer. Int J Radiat Oncol Biol Phys.

[47] Kirisits C, Pötter R, Lang S, Dimopoulos J, Wachter-Gerstner N, Georg D (2005). Dose and volume parameters for MRI-based treatment planning in intracavitary brachytherapy for cervical cancer. Int J Radiat Oncol Biol Phys.

[48] Datta NR, Srivastava A, Maria Das KJ, Gupta A, Rastogi N (2006). Comparative assessment of doses to tumor, rectum, and bladder as evaluated by orthogonal radiographs vs. computer enhanced computed tomography-based intracavitary brachytherapy in cervical cancer. Brachytherapy.

[49] Cheng JC, Peng LC, Chen YH, Huang DY, Wu JK, Jian JJ (2003). Unique role of proximal rectal dose in late rectal complications for patients with cervical cancer undergoing high-dose-rate intracavitary brachytherapy. Int J Radiat Oncol Biol Phys.

[50] Morris M, Eifel PJ, Lu J, Grigsby P, Levenback C, Stevens RE (1999). Pelvic radiation with concurrent chemotherapy compared with pelvic and para-aortic radiation for high-risk cervical cancer. New Engl J Med.

[51] Haie-Meder C, de Crevoisier R, Bruna A, Lhommé C, Pautier P, Morice P (2005). Concomitant chemoradiation in patients with cervix cancer. Bull Cancer.

[52] Heinzelmann F, Henke G, von Grafenstein M, Weidner M, Paulsen F, Staebler A (2012). Adjuvant radiochemotherapy in patients with locally advanced high-risk cervical cancer. Strahlenther Onkol.

[53] Kim JY, Kim JY, Kim JH, Yoon MS, Kim J, Kim YS (2012). Curative chemoradiotherapy in patients with stage IVB cervical cancer presenting with paraortic and left supraclavicular lymph node metastases. Int J Radiat Oncol Biol Phys.

[54] Perez CA, Grigsby PW, Castro-Vita H, Lockett MA (1995). Carcinoma of the uterine cervix. I. Impact of prolongation of overall treatment time and timing of brachytherapy on outcome of radiation therapy. Int J Radiat Oncol Biol Phys.

